# Crop Sorghum Ensiled With Unsalable Vegetables Increases Silage Microbial Diversity

**DOI:** 10.3389/fmicb.2019.02599

**Published:** 2019-11-15

**Authors:** Daniel L. Forwood, Kristian Hooker, Eleonora Caro, Yuxin Huo, Devin B. Holman, Sarah J. Meale, Alex V. Chaves

**Affiliations:** ^1^School of Life and Environmental Sciences, Faculty of Science, The University of Sydney, Camperdown, NSW, Australia; ^2^School of Agriculture and Food Sciences, Faculty of Science, The University of Queensland, Gatton, QLD, Australia; ^3^Department of Agricultural, Forestry and Food Science, University of Turin, Turin, Italy; ^4^Lacombe Research and Development Centre, Agriculture and Agri-Food Canada, Lacombe, AB, Canada

**Keywords:** 16S rRNA gene sequencing, fungal ITS sequences, reducing food waste, *in vitro* rumen fermentation, alternative livestock feeds

## Abstract

Ensiling vegetables with forage crops is a suggested method of waste diversion and can be directly utilized as a livestock feed. Carrot or pumpkin, ensiled at 0, 20, or 40% dry matter (DM) with crop sorghum, and with or without a second-generation silage inoculant were assessed for nutritive composition, organic acid profiles, aerobic stability and *in vitro* rumen fermentation characteristics. The study was a completely randomized design, with the fixed effects consisting of vegetable type (carrot vs. pumpkin), level (i.e., the level of vegetables), inoculant (inoculant or non-inoculant) and the interactions, and mini-silos within treatment as the random effect. The experimental unit for sorghum treatments represented by each mini-silo (5 kg capacity). Silage was sampled after 70-days ensiling for nutrient composition, 14-day aerobic stability, organic acid profiles and microbial diversity. After 24 h *in vitro* incubation, rumen fermentation parameters were assessed, measuring gas and methane (CH_4_) production, *in vitro* digestibility and volatile fatty acid concentrations. Sorghum ensiled with carrot or pumpkin at 20% or 40% DM increased crude fat (*P* ≤ 0.01) and decreased (*P* ≤ 0.01) silage surface temperature upon aerobic exposure compared to the control. Bacterial communities analyzed through 16S rRNA gene sequencing linearly increased (*P* ≤ 0.01) in diversity across both vegetables when the vegetable proportion was increased in the silage; dominated by *Lactobacillus* species. ITS analysis of the fungal microbiota upon silage opening and after 14 days (aerobic stability) identified increased (*P* ≤ 0.03) fungal diversity with increasing vegetable proportions, predominantly populated by *Fusarium denticulatum*, *Issatchenkia orientalis*, *Kazachstania humilis*, and *Monascus purpureus*. Upon assessment *in vitro*, there was an increase (*P* ≤ 0.04) in *in vitro* digestibility and some CH_4_ parameters (% CH_4_, and mg CH_4_/g DM), with no effect (*P* ≥ 0.17) on remaining CH_4_ parameters (mL CH_4_/g DM, mg CH_4_/g digested DM), gas production or pH. However, increasing vegetable amount decreased percentage of acetic acid and increased percentage of propionic acid of the total VFA, decreasing A:P ratio and total VFA concentration as a result (*P* ≤ 0.01). The results from this study indicate including carrot or pumpkin at 20 or 40% DM in a sorghum silage can produce a highly digestible, microbially diverse and energy-rich livestock feed.

## Introduction

The discarding of fruit and vegetables during processing in Australia and other nations accounts for 20% of total losses in production or harvest (Panda et al., 2016). As the issue of food security grows over the 21st century, alternative methods to utilize unsalable vegetables destined for landfill have been of recent interest. One such method is the production of ruminant feeds from these vegetables, replacing conventional disposal methods of landfill or composting. One of the main concerns when feeding discarded vegetables is their rapid expiration (i.e., short shelf-life) resulting from a high moisture content, thus contributing to greenhouse gas (GHG) emissions during decomposition (Sagar et al., 2018). As such, the conservation of vegetables through ensiling has been considered as a method to conveniently prolong the shelf-life of discarded vegetables (Bakshi et al., 2016).

Production of a uniform, high-quality silage is dependent on several factors, including crop and crop maturity, fiber chop length and the strain of lactic acid bacteria (LAB) inoculant (Tabacco et al., 2011; Borreani et al., 2018). Sorghum is a high-yielding, drought-tolerant crop well suited to Australian climates. Forage, grain and sweet sorghum crops are commonly used in fodder production, with reported crude protein content ranging between 6.64 and 11.71% (Behling Neto et al., 2017). This is comparatively lower than barley (12.5%) and oat (14.1%) silages (González-García et al., 2016). Addition of vegetables such as carrot, comprising 9.83% DM crude protein (Singh et al., 2001) to sorghum forage could increase digestibility, nutrient availability and accelerate the attainment of livestock growth targets. However, an evident knowledge gap arises regarding the fermentation profiles, nutritive characteristics and microbial profiles of silages containing unsalable vegetables.

Fruits and vegetables are known to possess a high degree of epiphytic and endophytic microbial diversity. For example, carrots and pumpkins are, respectively, exposed to the rhizosphere and phyllosphere (Jackson et al., 2015). The abundance and diversity of epiphytic bacterial communities present on the surfaces of fresh produce (Leff and Fierer, 2013) may increase their suitability as candidate additives for ensiling. However, at processing, vegetables are washed with pathogen-inactivating chemicals such as sodium hypochlorite (NaClO) or chlorine (Cl) (Haute et al., 2013), reducing surface microbial diversity. Despite this, it is suggested that the endophytic population of root vegetables such as carrots can remain relatively intact after chemical treatment, as bacteria persist within the cells of the vegetable tissue (Zhao et al., 2015).

The objective of this study was to evaluate the influence of ensiling fresh, unsalable carrots or pumpkin with crop sorghum, with or without a LAB inoculant, on physio-chemical composition, organic acid concentration, aerobic stability, gas production, fermentation characteristics *in vitro*, and microbial diversity. It was hypothesized that ensiling fresh, unsalable carrots [9.7% DM, 4.8% crude protein (CP), 11.1% neutral detergent fiber (aNDFom)] or pumpkin (12.6% DM, 10.7% CP, 18.1% aNDFom) with crop sorghum would elicit greater dry matter (DM) digestibility and microbial diversity than a control silage, with the introduction of a second-generation inoculant improving the aerobic stability of the vegetable silage. It was also hypothesized that the vegetable silages would have an inhibitory effect on CH_4_ production and increase rumen fermentation end-products.

## Materials and Methods

### Silage Production

The sorghum crop was collected during harvest to a DM content of approximately 35% and neutral detergent fiber (aNDFom) of 49.5% (DM-basis) from the University of Queensland (Gatton, QLD 27°56′ S, 152°28′ E) in January 2018. Vegetables (carrot and pumpkin; 9.7% and 12.6% DM content, 4.8% and 10.7% crude protein, 11.1% and 18.1% aNDFom, and 5.1% and 6.2% ash, respectively) unsuitable for commercial use were obtained from Kalfresh, Kalbar QLD (27°94′ S, 152°57′ E). Grevillea Ag supplied the second-generation silage inoculant (SI-LAC^®^ EXTRA) containing homolactic bacteria (*Lactobacillus plantarum* and *Enterococcus faecium*) and heterolactic bacterial species (*Lactobacillus buchneri*). Inoculant dose used was as indicated on the sachet; 10 g per 1.1 t of crop (lot number E563; Manufactured Dec 2017 and valid until Dec 2020). An appropriate amount of SI-LAC^®^ EXTRA product was dissolved in 25 mL of distilled water and applied to 10 kg of freshly chopped sorghum forage with or without unsaleable vegetables (*n* = 2 mini-silos per day per treatment) using a hand sprayer. Inoculant was mixed with the sorghum crops with or without unsaleable vegetables using a polyethylene trap (2.4 × 3.0 m) for a period of 2 min after application. The trap was sprayed with 70% ethanol and wiped with paper towel between treatments to avoid microbial contamination among the treatments.

The sorghum was harvested over 2 days, resulting in two mini-silos per day (10 treatments × 2 mini-silos per day = 20 mini-silos were packed in each day) and consequently four mini-silos per treatment. A total of 40 mini-silos were during the experiment. These mini-silos were produced from PVC piping and fitted with an airlock bubbler, measuring 90 mm diameter by 55 cm height and a volume of approximately 3500 cm^3^ and 5 kg capacity. Silos were weighed before and after filling and then stored at ambient temperature. A hydraulic press was used to pack each mini-silo to densities of approximately 240 kg/m^3^. On a DM basis, treatments consisted of 100% crop sorghum (control), 20% carrot with 80% crop sorghum; 20% pumpkin with 80% crop sorghum; 40% carrot with 60% crop sorghum and 40% pumpkin with 60% crop sorghum. Before packing, samples were obtained to determine initial bacterial microbiota present on the surface of the sorghum crop. Replicates of the treatments were produced, for comparison between inoculated and uninoculated silage. Further, exact weights of the mini-silos were measured and recorded at sealing and after 70 days ensiling.

### Opening of Mini-Silos

#### Silage Sampling

Mini-silos were weighed and recorded prior to aperture after an ensiling period of 70 days. Approximately 10 cm of silage from the top of the mini-silo was disposed of due to spoilage. Contents of each mini-silo was mixed with 100 g random samples placed into liquid nitrogen and stored at −20°C for DNA analysis (*n* = 4 DNA samples per treatment).

#### Dry Matter Content and DM Loss

An estimated 290 g of silage from each mini-silo were added to two aluminum trays per treatment for DM content analysis. The trays were dried at 55°C and removed after 48 h for weight measurement. Calculation of DM loss was through the difference between wet weight at the time of ensiling and at aperture (Zahiroddini et al., 2004).

#### pH

Approximately 15 g of silage from each mini-silo was sampled and combined with 135 g distilled water for a 1:10 dilution and blended at room temperature for 30 s (Zahiroddini et al., 2004). The solution was then filtered through double-layered cheesecloth. Approximately 15 mL of filtrate was collected, mixed and immediately measured via a pH meter (Activon Model 209, Gladesville, NSW, Australia) probe for pH value.

### Organic Acids, VFA, and Ethanol

The filtrate was sub-sampled, a collection of 30–40 mL poured into a 50 mL centrifuge tube and placed on ice for organic acids and volatile fatty acids (VFA) analyses. The organic acids and VFA concentrations were determined via the methods described by Playne (1985) post-collection of filtrates from mini-silos. Samples were centrifuged at 10,000 × *g* for 15 min at 4°C. A volume of 5 mL supernatant was collected from each sample and combined with 1 mL of 25% (wt/vol) metaphosphoric acid at a ratio of 5:1. Samples were frozen at −20°C, until VFA determination on an Agilent technologies 7820A gas-liquid chromatograph system, using a DB-FFAP column of dimensions 30 m × 0.32 mm × 1.00 μm, installed with a flame ionization detector (FID) set up at 250°C, air flow 350 mL/min, H_2_ fuel flow 30 mL/min, makeup flow (N_2_) 30 mL/min Split Inlet heated to 225°C, 9.526 PSI, Helium total flow 33 mL/min, septum purge flow 3 mL/min, split ratio 5:1, Split Flow 25 mL/min. Oven temperature was set to 150°C and held for 1 min, then 5°C per minute up to 195°C and sustained for 3 min. Organic acids (lactic and succinic) GC set up: oven temperature set to 45°C (held 1 min), then 30°C per min to 150°C, then 5°C per min to 190°C. Splitless inlet set at 190°C, FID 250°C (H_2_ = 30 mL/min, Air = 350 mL/min, N_2_ = 25 mL/min), carrier (He) at constant flow ∼ 1.5 mL/min. Ethanol GC set up: Oven temperature 80°C (hold 3 min), 25°C/min to 200°C (hold 1 min); Splitless inlet 225°C; FID 260°C (H_2_ = 30 mL/min, Air = 350 mL/min, N_2_ = 25 mL/min); carrier (He) at constant flow ∼ 1.5 mL/min. Malonic acid (50 mM) was used as an internal standard for organic acids and ethanol analyses. The concentrations of organic acids and VFA were expressed in mM and ethanol expressed as %.

### Aerobic Stability

Aerobic stability for each silage sample was measured by collection of 500 g of silage per mini-silo into two aluminum trays and exposure to ambient temperature for 14 days. A FLIR E50 Thermal Imaging Camera (FLIR, Wilsonville, OR, United States) and FLIR Tools software was used to obtain thermal images of each silage sample tray, taken from 1 m for 14 consecutive days at 0900 and 1600. A total of 30 thermal images per tray were taken from day 0 through day 14. Visual assessment of microbial spoilage and photographic estimates of the mean, minimum and maximum temperatures for each tray per day were recorded. At the completion of the 14-day period, silage samples (e.g., one sample per mini-silo) were mixed thoroughly, and 70 g samples were collected for DNA extraction of aerobically exposed silage. These samples were frozen with liquid nitrogen and stored at −20°C.

### DNA Extraction

DNA was extracted from silage samples (one sample per mini-silo) through repetitive bead beating, as described by Yu and Morrison (2004). Briefly, silage (∼300 mg) was weighed into a sterile 2 mL microcentrifuge tube, inclusive of 300 mg of 0.5 mm and 0.1 mm silica beads, 1.1 mL InhibitEx buffer was added, and samples were subjected to bead beating, incubation and centrifugation. The supernatant (600 μL) was then transferred into a sterile 2 mL microcentrifuge tube, along with 25 μL Proteinase-K. Samples were loaded into a QIAcube (Qiagen, Hilden, Germany) and analyzed for DNA yield through use of a NanoDrop (Thermo scientific NanoDrop Products) as described by Desjardins and Conklin (2010). The purity and integrity of DNA were quantified through methods described by Henderson et al. (2013).

### Microbial Community Analysis

#### Sequencing and Analysis of the 16S rRNA Gene and ITS Region

The V4 region of the archaeal and bacterial 16S rRNA gene was amplified as previously described by Terry et al. (2018), utilizing the 515f Modified (5′-GTGYCAGCMGCCGCGG TAA-3′) and 806r Modified (5′-GGACTACNVGGGTWTC TAAT-3′) primer sequences (Walters et al., 2016). The primers ITS1F (5′-CTTGGTCATTTAGAGGAAGTAA-3′) and ITS2 (5′-GCTGCGTTCTTCATCGATGC-3′) (Buée et al., 2009) were used to amplify the ITS1 region of fungi. Both 16S rRNA gene and ITS1 sequences were sequenced using the MiSeq Reagent Kit v2 (500 cycles; Illumina, Inc., San Diego, CA, United States) and an Illumina MiSeq instrument as per manufacturer’s instructions.

DADA2 v. 1.8 (Callahan et al., 2016) was used in R v. 3.5.1 to process the 16S rRNA gene and ITS1 sequences. Briefly, forward and reverse 16S rRNA gene sequences were trimmed to 220 and 200 bp, respectively, merged, and then chimeras were removed. Taxonomy was assigned to the remaining sequences, referred to here as operational taxonomic units (OTUs) at 100% similarity, using the RDP naïve Bayesian classifier and the SILVA SSU database release 132 (Quast et al., 2012). For ITS1 sequences, reads were quality-filtered using the default parameters and a minimum length of 50 bp but were not trimmed to the same length. The reads were merged, chimeras removed, and taxonomy assigned to the ITS1 sequences using the RDP naïve Bayesian classifier and the UNITE database v. 8.0 (Kõljalg et al., 2013). The number of OTUs per sample, Shannon diversity index, and inverse Simpson’s diversity index for 16S rRNA gene and ITS1 datasets were calculated in R using Phyloseq v. 1.26.0 (McMurdie and Holmes, 2013). Bray–Curtis dissimilarities were calculated using vegan 2.5-3 (Oksanen et al., 2013) in R and the effect of vegetable addition to the silage and inoculant was assessed using PERMANOVA. The silage 16S, silage ITS1, crop 16S, and the aerobic stability ITS1 samples were randomly subsampled to 4,450, 2,000, 1,000, and 6,000 sequences per sample, respectively, prior to analysis of alpha- and beta- diversity. All 16S rRNA gene and ITS1 sequences were submitted to the sequence read archive under BioProject accession PRJNA525850.

### *In vitro* and Rumen Fermentation Characteristics

#### *In vitro* Incubations

The *in vitro* study was conducted at the University of Queensland (Gatton, QLD, Australia). Steers used in this study were cared for under the approval and guidance of The University of Queensland Animal Ethics Committee (Approved Protocol Number AE35581). Two rumen-cannulated Droughtmaster steers were fed a diet including *ad libitum* pasture supplemented daily with 3 kg carrot and pumpkin, respectively (up to a maximum of 30% DM) for 1 week prior to the *in vitro* study. Rumen fluid was collected from the dorsal, anterior ventral, medium ventral, posterior dorsal and posterior ventral regions of the rumen at 2 h post-feeding (Meale et al., 2012, 2013). Rumen fluid from both steers were pooled, strained through four layers of cheesecloth into a pre-warmed, insulated 1 L Thermos and returned to the laboratory immediately. Rumen inoculum was prepared using the mixture described by Menke et al. (1979). ANKOM bags containing 0.5 g DM of each treatment were placed into 50 mL amber bottles prior to incubation. The bottles were pre-warmed for an hour prior in a water bath at 39 ± 0.5°C, then promptly gassed under a stream of O_2_-free N_2_ gas. The bottles were filled with 25 mL inoculum and fitted with rubber stoppers to prevent the escape of gases produced during fermentation. Bottles were then replaced into the shaking water bath at 90 oscillations per minute. Blank samples included three bottles containing only 25 mL inoculum. The *in vitro* incubations were repeated twice, with two replicates of each mini-silo per treatment for the 24 h sampling interval.

#### CH_4_, Total Gas, and *in vitro* Dry Matter Disappearance

After 24 h incubation, 17 mL gas samples were extracted and inserted into the rubber stopper, with an estimated 10 mL gas transferred to 6.8 mL evacuated Exetainers (Labco Ltd., High Wycombe, United Kingdom) for CH_4_ analysis. Measurement of CH_4_ concentration occurred through use of an Agilent model 7890a gas chromatograph with a flame ionization detector (FID) calibrated to 250°C, air flow 300 mL/min, H_2_ fuel flow 30 mL/min, makeup flow (N_2_) 30 mL/min installed with a capillary column (Restek Rt-Q-Bond, 30 m × 0.53 mmID × 20 μm). The Split-Splitless Inlet was heated to 60°C, 9.526 PSI, with Helium total flow 33 mL/min, septum purge flow 3 mL/min, Split ration 5:1, Split Flow 25 mL/min and an oven temperature of 60°C. Methane measurements were defined as mg CH_4_/g DM and mg CH_4_/g digested DM.

After 24 h, total gas production was measured immediately after sampling for CH_4_ concentration using a water displacement apparatus (Fedorah and Hrudey, 1983). Total gas production and pH were measured through methods described by Meale et al. (2012). To reduce the likelihood of continued fermentation, ANKOM bags were removed from the bottles and placed on ice. The ANKOM bags were rinsed thoroughly with distilled water using the ANKOM Fiber Analyzer (two cycles at 10 min and 100°C), dried at 55°C for 48 h to constant weight and weighed to estimate *in vitro* dry matter disappearance (IVDMD).

#### VFA Analysis

Contents of the incubation bottles were transferred into 2 mL microcentrifuge tubes. At 4,500 × *g* for 15 min at 5°C, samples were centrifuged for VFA analysis. The supernatant was subsampled (1.5 mL) and transferred into 2 mL microcentrifuge tubes, then acidified with 0.3 mL of metaphosphoric acid (0.20; w/v). Subsamples were frozen at −20°C until analyzed for VFA concentrations using a gas chromatograph (Please see section “Organic Acids, VFA, and Ethanol” for details). The concentration of total VFA was expressed in mM and individual VFA expressed as% of total VFA (mmol/100 mmol).

#### Chemical Analysis

The DM content of silage samples was determined by oven-drying at 55°C for 72 h. Dried samples were ground through a 1-mm screen and analyzed for neutral detergent fiber (NDF) as described by Van Soest et al. (1991), modified for an Ankom 200/220 Fiber Analyzer (Ankom Technol. Corp., Fairport, NY, United States). Amylase and sodium sulfite were included in the NDF analyses and is expressed inclusive of residual ash (aNDFom). Ash content was determined after 2 h of oxidation at 600°C in a muffle furnace (AOAC, 1990 method 942.05). Nitrogen was quantified by combustion method (method 990.03). Crude protein (CP) content, calculated as N × 6.25, was applied for nitrogen (N) concentration determination. Crude fat was determined by extraction with ether as described for lipid extraction (method 920.29; AOAC, 1990), modified for an Ankom Fat Analyzer (Ankom Technol. Corp., Fairport, NY, United States). Non-fibrous carbohydrate (NFC) was calculated as by Mertens (2002):

NFC=100-CP-aNDFom-crude⁢fat-ash

### Statistical Analysis

Statistical analyses of data recorded for nutrient, organic acid composition and aerobic stability were conducted through PROC MIXED SAS (SAS Institute Inc, 2019), with results presented as least squares mean and standard error of the mean (SEM). The data was analyzed as a completely randomized design, with the fixed effects consisting of vegetable type (carrot vs. pumpkin), level (i.e., the level of vegetables on a DM basis in the sample; 0, 20 and 40%), inoculant (inoculant or non-inoculant) and the interactions, and mini-silos within treatment as the random effect. As the sorghum silage was harvested over 2 days, there were four replicates per treatment resulting in a total of 40 mini-silos, the experimental unit for sorghum treatments represented by each mini silo.

Data were assessed for normality through PROC UNIVARIATE SAS. Orthogonal polynomial contrasts were also used to evaluate linear and quadratic responses to varied addition of carrots or pumpkins (0, 20, and 40% on a DM basis) in the various silage treatments. Differences among means were determined using a least square linear hypothesis test, with the significance for all data declared at *P* ≤ 0.05.

Differentially abundant OTUs among silage mixtures were determined using DESeq2 v. 1.22.1 (Love et al., 2014). For this analysis, non-subsampled OTU tables were used but only OTUs found in at least 25% of the samples analyzed were included. *In vitro* data were analyzed under the LSMEANS/DIFF function procedure in SAS using treatment means (vegetable, level, inoculant and a combination of interactions) as the fixed effects, run, run × vegetable treatment and level × inoculant interactions as random effects with run × treatment as an error term, testing the treatment effect.

## Results

### Silage Chemical Composition

As the level of carrot or pumpkin inclusion increased on a DM basis in the silage, DM content decreased (Table 1; *P* ≤ 0.01) while there was no effect (*P* = 0.13) of the vegetable type × level interaction. The addition of 40% vegetables to the sorghum, yielded lower DM contents of 28.9% and 25.6% for carrots and pumpkin, respectively, compared to the control. The ash content of the sorghum silage was not affected (*P* ≥ 0.14) by vegetable type or the level of vegetable used (Table 1). However, the aNDFom percentage of the sorghum silage linearly decreased (*P* < 0.01) with increased levels of vegetable used. Crude fat also linearly increased (*P* ≤ 0.01) with increasing levels of inclusion of carrot or pumpkins (Table 1).

**TABLE 1:** Nutrient composition and organic acid profile of a sorghum and vegetable silage after 70 days of ensiling.

		**Carrots**		**Pumpkin**			***P*-value**								
	**0%**	**20%**	**40%**	**20%**	**40%**	**SEM**	**Veg**	**Level**	**Veg × Level**	**Inoculant**	**Veg × Inoculant**	**Level × Inoculant**	**Veg × Level × Inoculant**	**Linear**	**Quadratic**
Dry matter content, %	36.1	33.6	28.9	35	25.6	1.21	0.50	<0.01	0.13	0.77	0.34	0.39	0.50	<0.01	0.01
Crude protein (CP), % DM	5.7	5.5	8.5	5.7	7.8	n/a	n/a	n/a	n/a	n/a	n/a	n/a	n/a	n/a	n/a
Non-fibrous carbohydrates (NFC)^1^,% DM	34.9	34.7	34.9	35.8	34.9	n/a	n/a	n/a	n/a	n/a	n/a	n/a	n/a	n/a	n/a
Crude fat, % DM	2.9	4.1	4.5	3.8	4.3	0.32	0.54	<0.01	0.90	0.76	0.56	0.66	0.50	<0.01	0.33
Neutral detergent fiber (aNDFom), % DM	49.9	49.1	45.5	48.2	46.0	0.63	0.81	<0.01	0.58	0.08	0.79	0.09	0.96	<0.01	0.15
Ash, % DM	6.6	6.6	6.6	6.5	7.0	0.14	0.47	0.17	0.31	0.68	0.07	0.38	0.39	0.09	0.45
Dry matter loss, g	58.8	61.3	73.1	47.1	66.9	6.56	0.21	0.03	0.51	0.39	0.51	0.02	0.86	0.06	0.04
Silage pH	3.7	3.6	3.6	3.6	3.6	0.06	0.59	0.61	0.91	0.95	0.86	0.87	0.99	0.42	0.67
**Organic compounds**															
Lactic acid, mM	12.1	9.4	10.6	10.7	12.2	1.28	0.40	0.32	0.82	0.76	0.74	0.83	0.93	0.64	0.16
Succinic acid, mM	0.33	0.32	0.37	0.29	0.27	0.04	0.26	0.83	0.57	0.95	0.78	0.68	0.98	0.89	0.56
Ethanol, %	0.11	0.09	0.09	0.10	0.11	0.012	0.31	0.61	0.70	0.05	0.95	0.17	0.98	0.47	0.54
**Volatile fatty acids (VFA), mM**															
Acetic acid	7.8	8.0	9.1	9.0	9.4	0.78	0.52	0.20	0.83	0.37	0.65	0.53	0.92	0.08	0.96
Propionic acid	0.21	0.21	0.23	0.22	0.21	0.01	0.55	0.29	0.22	0.46	0.39	0.02	0.63	0.13	0.74
Butyric acid	0.08	0.06	0.12	0.07	0.08	0.01	0.10	<0.01	0.06	0.21	0.29	0.52	0.13	0.04	<0.01
Total VFA	8.6	8.7	9.7	9.6	10	0.78	0.51	0.28	0.83	0.36	0.65	0.53	0.92	0.11	0.97

*n/a, not-applicable; ^1^NFC = 100 - CP - aNDFom - crude fat - ash; SEM, standard error of the means.*

There was an increase (*P*_level × inoculant_ = 0.02) in DM loss (in g) following ensiling correspondent with increasing vegetable inclusion levels when inoculants were used (0% = 47.5b, 20%:58.1ab, 40% = 71.3a). No difference (*P* > 0.05) on DM loss with increasing level of vegetables when silage were not inoculated. Silage pH was not affected (*P* ≥ 0.10) by the addition of type or level of vegetables or inoculant in the sorghum silages.

### Volatile Fatty Acids and Organic Acid Concentrations

The concentrations of acetic, succinic and lactic acids were unaffected (*P* ≥ 0.20) by type of vegetable, level, inoculant and interactions (Table 1). The concentration of propionic acid in the sorghum silage was not affected (*P* ≥ 0.13) by vegetable or level but was affected (*P* = 0.02) by level × inoculant. This two-way interaction suggested the inclusion of inoculant led to an increase in propionic acid concentration as vegetable level increased. Further, the concentration of butyric acid in the sorghum silage also increased (*P* < 0.01) with the inclusion of 40% vegetable, compared to the control, with 40% carrot inclusion proving most effective at increasing the butyric acid concentration from 0.08 mM to 0.12 mM (Table 1). However, total VFA concentration was not affected (*P* ≥ 0.11) by vegetable type, level, inoculant or interactions in the sorghum silages. Ethanol concentration was greater (*P* = 0.05) within non-inoculant silages (0.11%) compared to inoculant silages (0.09%). Vegetable type, level and interactions did not affect ethanol concentration in the sorghum silages.

### Silage Aerobic Stability

**FIGURE 1:**
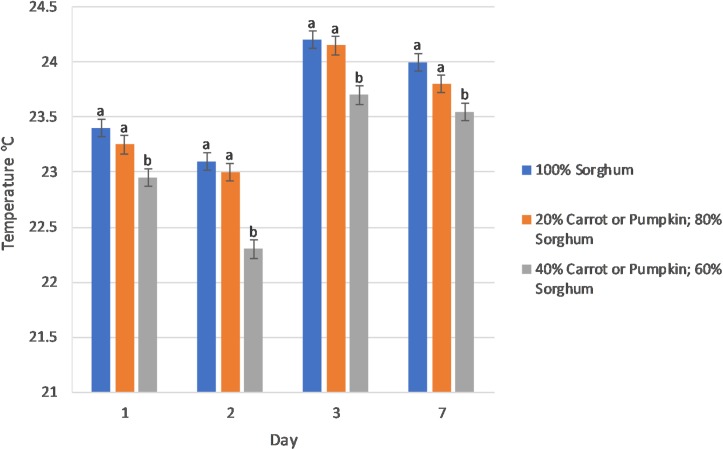
The level of vegetables (0, 20, and 40% on a dry matter basis) in the sorghum silage affected (*P* < 0.01) the surface temperature (°C) of sorghum vegetable silage during aerobic stability at days 1–3 and 7. The fixed effects and interactions Vegetable type (Veg), Veg × Level, Inoculant, Veg × Inoculant, Level × Inoculant, Veg × Level × Inoculant were *P* > 0.10.

The aerobic stability of sorghum silage was positively affected (*P* < 0.01) by an increase in vegetable content exhibiting a linear (*P* ≤ 0.04) decrease in the surface temperature of the silage as the vegetable concentration increased over multiple days (Figure 1). The change in surface temperature of the sorghum silage was affected (*P* ≤ 0.04) by the level of vegetables in the mini-silos on 10 of the 14 days of the aerobic stability trial (Supplementary Figure S1). Furthermore, 11 of the 14 days expressed an increasing linear (*P* ≤ 0.03) relationship, whereby as the level of vegetable increased, so too did the amount of surface temperature change (°C) of the silage. In addition, the use of inoculant decreased surface temperature (*P* = 0.03) on day 6 of the trial (data not presented).

### Silage Bacterial and Fungal Composition and Diversity

The alpha diversity metrics (within sample richness and diversity) for crop and silage microbiota are presented in Table 2. Increasing the concentration of vegetables decreased (*P* < 0.01) the number of bacterial OTUs in the sorghum crop before the ensiling process. With the addition of a silage inoculant, the Shannon and inverse Simpson’s diversity indices decreased (*P* ≤ 0.04) by 12.6 and 66.4%, respectively, when compared to the sorghum crop without inoculant. Bacterial diversity indexes were affected by the vegetable type × level interaction (Table 2). Addition of carrots at 40% DM increased (*P* ≤ 0.01) the number of OTUs and Shannon diversity compared to the other treatments. The 100% control sorghum silage and sorghum silage with 20% DM carrots had a lower (*P* < 0.01) inverse Simpson’s diversity index compared to other treatments.

**TABLE 2:** Bacterial and fungal operational taxonomic unit (OTU) alpha diversities of sorghum silage with increasing proportions of vegetables.

		**Carrots**		**Pumpkin**			***P*-value**								
	**0%**	**20%**	**40%**	**20%**	**40%**	**SEM**	**Veg**	**Level**	**Veg × Level**	**Inoculant**	**Veg × Inoculant**	**Level × Inoculant**	**Veg × Level × Inoculant**	**Linear**	**Quadratic**
**16S RNA – Original crop**															
Number of OTUs	48.6	33.2	35.7	22.0	35.9	15.20	0.37	<0.01	0.32	0.27	0.16	0.42	0.48	0.04	<0.01
Shannon diversity	2.31	2.22	2.21	1.80	2.33	0.76	0.43	0.06	0.15	0.05	0.18	0.44	0.54	0.84	0.03
Simpsons diversity	7.98	7.02	6.80	4.39	7.00	4.09	0.44	0.11	0.38	<0.01	0.61	0.04	0.92	0.50	0.10
**16S RNA – Silage**															
Number of OTUs	41.1b	58.4b	92.4a	46.8b	48.1b	6.49	<0.01	<0.01	0.01	0.29	0.51	0.51	0.75	<0.01	0.58
Shannon diversity	2.63b	2.62b	3.24a	2.52b	2.53b	0.102	<0.01	0.01	<0.01	0.59	0.95	0.85	0.75	0.02	0.03
Simpsons diversity	9.50b	8.35b	15.52a	6.97a	7.38a	0.738	<0.01	<0.01	<0.01	0.97	0.97	0.30	0.93	0.08	<0.01
**ITS – Silage**															
Number of OTUs	33.6	40.6	31.3	37.5	30.6	10.13	0.69	0.13	0.91	0.02	0.68	0.87	0.62	0.51	0.05
Shannon diversity	2.79	2.83	2.09	2.65	2.52	0.362	0.58	0.03	0.25	0.09	0.64	0.15	0.70	0.01	0.22
Simpsons diversity	11.6	10.7	6.6	9.3	10.0	2.76	0.65	0.22	0.42	0.60	0.87	0.12	0.72	0.09	0.99
**ITS – Aerobic stability**															
Number of OTUs	22.5	17.0	36.0	31.0	56.0	5.07	0.01	<0.01	0.13	0.99	0.27	0.02	0.74	<0.01	0.02
Shannon diversity	1.55	1.12	1.92	1.75	2.86	0.305	0.03	<0.01	0.24	0.39	0.33	0.03	0.73	0.01	0.03
Simpsons diversity	3.90	2.33	5.47	4.48	12.67	1.519	0.02	<0.01	0.07	0.36	0.88	0.19	0.57	<0.01	0.02

*SEM, standard error of the means.*

The number of fungal OTUs increased (*P* = 0.02) by 25% with the addition of an inoculant (Data not presented). In silages, vegetables added at 40% decreased (*P* = 0.03) the fungal Shannon diversity index by 16%, compared to 0 or 20%. However, there was no effect (*P* ≥ 0.12) on the fungal inverse Simpson’s diversity index due to vegetable type, concentration or inoculant inclusion. Moreover, fungal richness and diversity was consistent across the three indices. After 14-d of aerobic exposure, addition of pumpkin resulted in a greater (*P* ≤ 0.025) number of OTUs and higher Shannon and inverse Simpsons diversities compared to addition of carrots (Table 2). Overall, vegetables added at 40% (DM-basis) decreased the number of OTUs, Shannon and inverse Simpson’s diversities, compared to 0 or 20% after 14-days of aerobic exposure.

**FIGURE 2:**
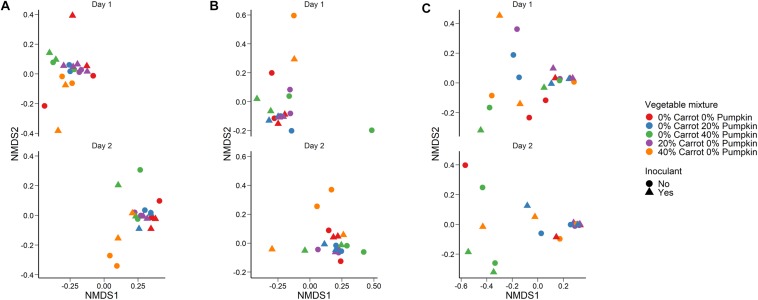
Non-metric dimensional scaling (NMDS) plot of the Bray–Curtis dissimilarities for the **(A)** bacterial microbiota in sorghum silage (Stress = 0.12), **(B)** fungal microbiota (Stress = 0.13) in sorghum silage, and **(C)** aerobic stability after 14 days aerobic exposure (Stress = 0.14) by vegetable mixture, sampling day, and use of an inoculant.

**FIGURE 3:**
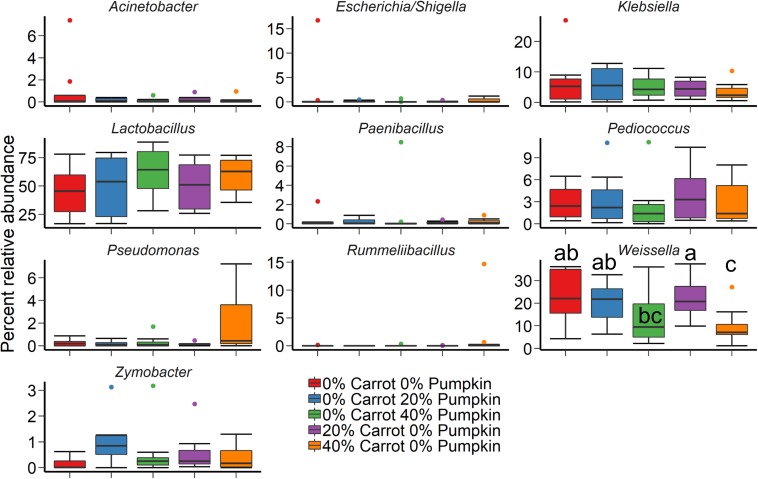
The 10 most relatively abundant bacterial genera in the sorghum silage after 70 days ensiling by vegetable mixture at 0, 20, or 40% DM. Different lowercase letters indicate significantly different means (*P* ≤ 0.05).

Crop sorghum, prior to ensiling was harvested on two different days. Sampling day (*R*^2^ = 0.44; *P* < 0.001) had a larger effect on the structure of the silage bacterial microbiota than vegetable mixture used (Figure 2A; *R*^2^ = 0.14; *P* < 0.001) based on Bray–Curtis dissimilarities and the PERMANOVA. The use of an inoculant had no significant effect on the sorghum silage microbiota (*R*^2^ = 0.02; *P* > 0.05). The sorghum silage bacterial microbiota was dominated by *Lactobacillus*, regardless of vegetable mixture (Figure 3). Members of the *Klebsiella*, *Pediococcus*, and *Weissella* genera were also relatively abundant in the silage (> 3%). We also identified differentially abundant bacterial OTUs between the 40% carrot or pumpkin silages and the 100% sorghum silage at both sampling days (Supplementary Table S1). Several OTUs classified as *Lactobacillus* were more abundant in the vegetable mixture silages, however, there were other *Lactobacillus* OTUs that were enriched in the 100% sorghum silage. This suggests that different *Lactobacillus* species may be more abundant in either the 100% or 40% vegetable silages. Additionally, a dominant *Pseudomonas* OTU was significantly more abundant (*P* < 0.05) in the 40% carrot silage at both sampling times.

**FIGURE 4:**
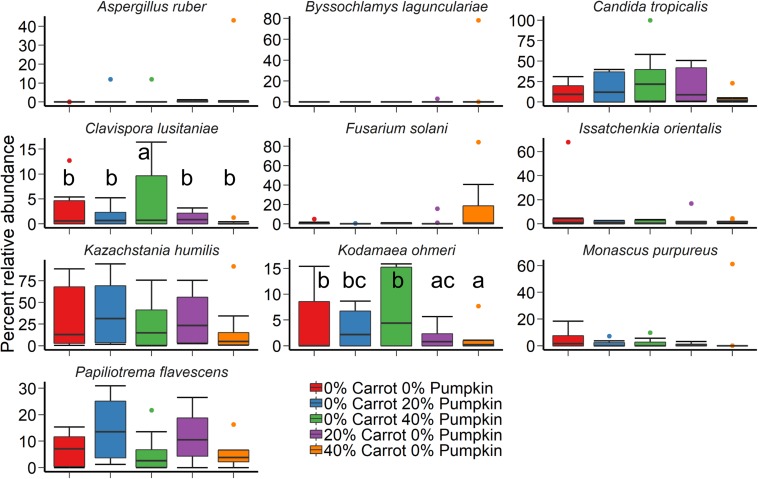
The 10 most relatively abundant fungal species in sorghum ensiled with carrot or pumpkin for 70 days, by vegetable mixture of carrots or pumpkin at 0, 20, and 40% DM. Different lowercase letters indicate significantly different means (*P* ≤ 0.05).

A large effect of day was also observed on the structure of the silage fungal microbiota (Figure 2B; *R*^2^ = 0.28; *P* < 0.001). Although there was considerable variability among samples, *Candida tropicalis*, *Kazachstania humilis*, and *Papiliotrema flavescens* were the most relatively abundant fungal species in the sorghum silage (Figure 4). Inclusion of 40% pumpkin increased (*P* < 0.05) the relative abundance of *Clavispora lusitaniae* compared to all other vegetable concentrations and 40% carrot reduced the relative abundance of *Kodamaea ohmeri* in relation to the 100% sorghum silage. The majority of silage samples that we sequenced had no detectable methanogens (< 0.02%) nor were they detected in any of the sorghum samples.

**FIGURE 5:**
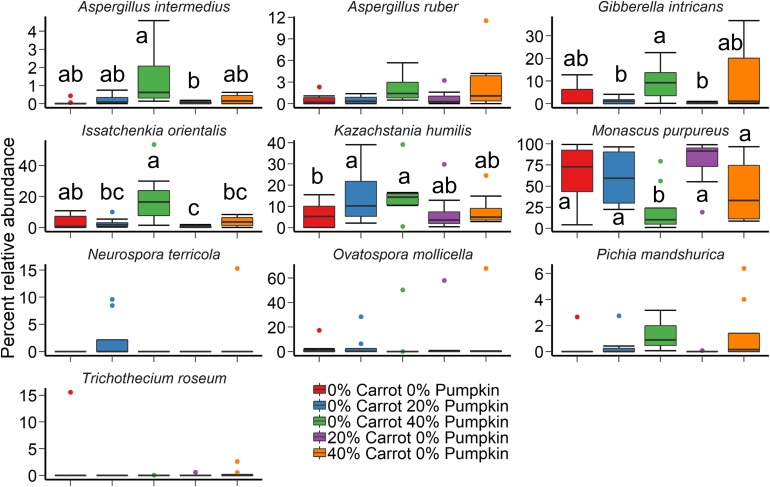
The 10 most relatively abundant fungal species in sorghum silage by ensiled vegetable mixture of carrot or pumpkin at 0, 20, or 40% DM after 14 days aerobic exposure. Different lowercase letters indicate significantly different means (*P* ≤ 0.05).

The vegetable mixture used in the sorghum ensiling process affected the fungal microbial community structure under aerobic exposure for 14 days (Figure 2C; *R*^2^ = 0.24; *P* < 0.01). The use of an inoculant had no effect (*P* > 0.05). For the sorghum silage aerobic stability, *Monascus purpureus*, *K*. *humilis*, and *Issatchenkia orientalis* were relatively abundant (>3%) in both aerobic stability and silage sampling (Figure 5). The relative abundance of *M. purpureus* and *K. humilis* was significantly lower in the 40% pumpkin silage aerobic stability samples compared with 100% sorghum (*P* < 0.05). Samples were also taken prior to ensiling to determine the initial bacterial microbiota present. The day that the sorghum was sampled had a strong effect on the initial bacterial community structure (*R*^2^ = 0.41; *P* < 0.0001) with day 2 samples appearing very similar to each other and day 1 samples more variable (Supplementary Figure S2). However, a number of samples were poorly sequenced due to the presence of plant material and had to be excluded from the analysis. For those samples with at least 1,000 16S rRNA gene sequences, the bacterial microbiota was largely dominated by *Klebsiella*, *Pseudomonas*, and *Weissella* (Supplementary Figure S3).

### *In vitro* Fermentation Characteristics

As the level of vegetables increased in sorghum silage DM, CH_4_ (%; mg/g DM) production also increased (*P* ≤ 0.04; Table 3). Inoculated silages had 10.62% greater (*P* < 0.05) CH_4_ production (mg/g DM) compared to non-inoculated silages. There were no further effects of vegetable inclusion on CH_4_ production parameters by vegetable type, concentration or their interaction. An effect was observed with IVDMD, in which a higher proportion of vegetable in the silage DM increased (*P* < 0.01) digestibility from 49.4% (0% vegetable) up to 57.1% (40% pumpkin). The same increasing effect was also observed for the Level × Inoculant interaction (*P* = 0.03) where inoculated sorghum silages with 40% vegetables had a 10.2% greater IVDMD compared to non-inoculated silage at the same vegetable level.

**TABLE 3:** The *in vitro* fermentation characteristics of sorghum silage ensiled with unsalable vegetables after 24 h incubation.

		**Carrots**		**Pumpkin**			***P*-values**								
	**0%**	**20%**	**40%**	**20%**	**40%**	**SEM**	**Veg**	**Level**	**Veg × Level**	**Inoculant**	**Veg × Inoculant**	**Level × Inoculant**	**Veg × Level × Inoculant**	**Linear**	**Quadratic**
Gas, mL/g DM	92.3	87.1	91.4	91.9	93.1	3.47	0.41	0.62	0.77	0.32	0.52	0.28	0.62	0.98	0.33
CH_4_,%	12.3	12.5	14.6	13.0	13.8	0.86	0.87	<0.01	0.54	0.06	0.47	0.18	0.39	<0.01	0.30
CH_4_, mg/g DM	8.4	8.4	9.6	9.2	9.9	0.70	0.39	0.04	0.76	<0.05	0.48	0.36	0.59	0.01	0.52
CH_4_, mg/g digested DM	17.1	16.5	17.9	17.8	17.3	1.32	0.81	0.85	0.70	0.07	0.46	0.13	0.60	0.62	0.81
pH	6.2	6.3	6.2	6.2	6.2	0.05	0.66	0.17	0.79	0.06	0.79	0.62	0.30	0.29	0.11
IVDMD	49.4	51.4	53.9	52.4	57.1	1.20	0.15	<0.01	0.39	0.35	0.94	0.03	0.57	<0.01	0.60
**Volatile fatty acids (VFA; mmol/100 mmol)**															
Acetic (A)	61.7	61.8	60.8	61.2	60.6	0.65	0.15	<0.01	0.46	0.64	0.59	0.40	0.27	<0.01	0.14
Propionic (P)	24.8	25.1	25.7	25.3	25.8	0.60	0.47	<0.01	0.78	0.63	0.61	0.20	0.09	<0.01	0.75
Butyric	11.1	10.7	11.0	11.0	11.1	0.20	0.32	0.38	0.68	0.25	0.69	0.86	0.95	0.87	0.19
Valeric	1.1	1.08	1.09	1.09	1.13	0.069	0.22	0.56	0.60	0.11	0.90	0.47	0.98	0.60	0.35
BCVFA	1.4	1.4	1.4	1.4	1.4	0.03	0.49	0.86	0.85	0.60	0.39	0.75	0.77	0.61	0.89
A:P ratio	2.5	2.5	2.4	2.4	2.4	0.08	0.26	<0.01	0.60	0.84	0.57	0.23	0.13	<0.01	0.44
Total VFA (mM)	88.3	90.4	93.3	90.8	93.8	3.53	0.72	<0.01	0.96	0.09	0.65	<0.01	0.93	<0.01	0.74

*SEM, standard error of the means; IVDMD, *in vitro* dry matter digestibility; BCVFA, branched-chain VFA (iso-butyric + iso-valeric).*

The percentage of propionate in the total VFA increased (*P* ≤ 0.01; Table 3) with vegetable inclusion at 20 or 40% into silage DM. However, the percentage of acetate decreased (*P* < 0.01) with the increase in inclusion levels of vegetable in the silage DM. These resultant increase (*P* ≤ 0.01) the acetic acid: propionic acid (A:P) ratio. There was a 4.4% increase (P_level × inoculant_ < 0.01) in total VFA concentration for inoculated silages with 40% vegetables compared to non-inoculated silages at the same vegetable level. No other effects were observed regarding a vegetable × level interaction.

## Discussion

### Silage Chemical Composition

Silages with low DM, thereby high moisture content are susceptible to seepage of nutrients, compromising silage quality and potentially leading to clostridial fermentation (Han et al., 2014). However, Garcia et al. (1989) reported that silages with 30–40% DM can be highly beneficial, reducing risk of heat-damage and increasing lactic acid fermentation. In this study, DM content of silages including vegetables were low, explained by the two-way interaction of level by inoculant. In this instance, DM loss was higher for mini-silos containing both vegetable and inoculant, than those without. This finding supports past reports of higher levels of DM loss during fermentation with the application of second-generation inoculants including *L. buchneri* (Kleinschmit and Kung, 2006; Jin et al., 2015). Kleinschmit and Kung (2006) demonstrated using laboratory-scale silages that the use of *L. buchneri* resulted in approximately 1% DM loss compared with untreated silage. Stoichiometrically, this microorganism breaks down 1 mole lactic acid → 0.48 mole acetic acid + 0.48 mole 1,2-propanediol + 0.04 mole ethanol + 0.52 mole CO_2_ (Oude Elferink et al., 2001).

### Organic Acid Concentrations

There was little influence of vegetable inclusion on the VFA concentrations produced through ensiling, with no statistical difference noted between acetic acid concentrations of carrot and pumpkin. A previous study by Zhang et al. (2016) identified variation between sorghum silage varieties and acetic acid concentration – forage sorghum at 15.63 mmol/L and high sugar sorghum 19.54 mmol/L. Variation was also confirmed in this study, through the lower concentration of acetic acid in the control silage (7.8 mmol/L). However, despite the lower acetic acid concentration in the sorghum silage, vegetable addition in this study yielded the highest concentration of acetic acid at 40% DM (9.4 mmol/L) probably due to the lower DM contents. Acetic acid has been proven to be the sole substance responsible for increased aerobic stability, and this acid acts as an inhibitor of spoilage organisms (Danner et al., 2003). Propionic acid is a by-product of the anaerobic degradation pathway of lactic acid (Elferink et al., 2001) which occurs in silages inoculated with *L. buchneri*. In the present study, the application of an inoculant increased propionic acid concentrations in inoculated sorghum silages as vegetable proportion increased compared to uninoculated silage. These findings are supported by studies that have identified higher propionic acid concentrations through use of *L. buchneri* compared to uninoculated samples (Elferink et al., 2001).

### Silage Aerobic Stability

In our study, a combination of increased vegetable level and a heterofermentative inoculant led to increased aerobic stability in silages containing 40% carrot or pumpkin. This was observed by lower recorded temperatures at 1, 2, 3, and 7-days aerobic exposure (Figure 1). Inoculating silage with *L. buchneri* has previously been associated with improving aerobic stability in sorghum-sudangrass silage (Li et al., 2016) through fermentation of lactic acid to acetic acid (Wilkinson and Davies, 2013), thus inhibiting yeast and mold proliferation (Silva et al., 2017). Our study further determined that increasing the proportion of vegetable, irrespective of vegetable type in an inoculated silage resulted in an increase in ethanol produced during ensiling. Regarded for its antimicrobial properties, ethanol has previously been associated with increased aerobic stability when used alongside LAB inoculants (Yuan et al., 2018).

### Microbial Community Analysis

The bacterial 16S rRNA gene and fungal ITS1 regions were sequenced to characterize the bacterial and fungal microbiota of sorghum ensiled with either carrots or pumpkin. Inclusion of carrots at 40% in the silage DM resulted in the greatest number of OTUs (richness) and the highest Shannon diversity values across all treatments. The bacterial silage microbiota was largely dominated by members of the *Lactobacillus* and *Weissella* genera. Wouters et al. (2013) reported that the initial fermentation of carrots in a mixed vegetable treatment also had an abundance of several bacterial genera identical to the vegetable silage in our study – namely *Lactobacillus* and *Weissella*. As fermentation progressed, these authors also identified *Pediococcus* and *Pseudomonas* spp. as prevalent in the 40% carrot treatment. However, *Lactobacillus* spp. dominated, regardless of treatment. The dominance of *Lactobacillus* in silage treatments is well known, and is compounded by the results from this study, whereby no effect was observed on microbial diversity indices through use of a silage inoculant. The higher bacterial Shannon diversity and the number of OTUs in the 40% carrot sorghum silage compared to all other treatments could be related to the root-based epiphytic population previously observed in carrots (Surette et al., 2003). Surette et al. (2003) analyzed carrots for epiphytic bacteria colonizing root systems and found 83% growth-promoting, 10% growth-neutral and 7% growth-inhibiting bacterial strains. Similar to our study, Surette et al. (2003) also detected *Pseudomonas* as being one of the most abundant bacterial genera. Therefore, it is postulated that the carrots in the 40% carrot silage treatment promote bacterial growth, hence increasing diversity. As the carrots for our study were sourced directly from a processing facility, the crown, the segment of carrot highest in sugar content (Surette et al., 2003) was relatively undisturbed, possibly benefiting the epiphytic population and enhancing bacterial richness and diversity in the sorghum vegetable silage.

Interestingly, the bacterial community structure was affected by sampling day. However, day of sampling had no effect on the relative abundance of *Lactobacillus* spp. in the silage, as it was among the most relatively abundant bacterial genera after 70 days ensiling. The dominance of *Lactobacillus* in a soybean-sorghum silage was observed by Ni et al. (2018), whereby 60–80% of the microbiota was comprised of *Lactobacillus* spp., followed by *Weissella* spp. As sorghum is high in WSC, it provides an environment conducive to a rapid decline in pH, followed by the proliferation of LAB upon fermentation (Guan et al., 2002).

Akin to the bacterial community, the fungal microbiota was also affected by sampling day. In agreement with our study, the proportion of *C. tropicalis* isolated in total mixed ration (TMR) and whole crop maize silages was reported by Wang et al. (2018) to be affected by the period of ensiling, decreasing in proportion when initial ensiling occurred. Upon exposure to aerobic conditions, *C. tropicalis* was not detected in samples due to elevated acetic acid concentrations, recorded as greater than 8 g/L (Wang et al., 2018). This has been corroborated by Ding et al. (2013), whereby high concentrations of acetic acid have inhibited gene expression and growth in industrial *Saccharomyces cerevisiae* strains. Conversely, increased utilization of lactic acid by fungi under aerobic conditions significantly influenced the fungal microbiota of sorghum vegetable silage. A relative abundance of *M. purpureus*, *K. humilis*, and *I. orientalis* was noted across both the silage and aerobic stability samples. Isolates of *I*. *orientalis* have previously been shown to utilize lactic acid, maltose and galactose as a substrate for growth in high moisture maize silage (Santos et al., 2017). This could pose an issue in industry, as prolonged aerobic exposure and the resultant proliferation of yeasts such as *I. orientalis* have decreased NDF digestion in *in vitro* studies (Santos et al., 2015).

### Rumen Fermentation Characteristics

By increasing the proportion of vegetable in the silage at 20 and 40% DM, CH_4_ parameters (%, mg/g DM) were increased, denying our initial hypothesis. Methods for reducing CH_4_ emissions in ruminant production have been an active area of investigation, with many studies placing emphases on dietary mitigation strategies as a means of CH_4_ management. Further, it should be noted that sorghum vegetable silages were found to linearly increase CH_4_ production, with inoculants having greater CH_4_ outputs compared to non-inoculated silages (Table 3). This result has not been observed in prior studies utilizing inoculants containing *L. plantarum*, a reduction in overall CH_4_ production instead observed at the completion of incubation (Ellis et al., 2016). Comprising a fraction of total gas produced, CH_4_ (mg/g DM incubated) in this study increased with inclusion of 40% carrots or pumpkin on a DM basis. Consequently, this increase can be attributed to the high digestibility, thus availability of vegetables for ruminal fermentation, as IVDMD increased with increasing vegetable proportion. Further, a greater quantity and faster rate of gas production have previously been related to increased IVDMD during *in vitro* fermentation of vegetables, whereby carrots and squash displayed increased gas production alongside orange and peppers (Marino et al., 2010). In this instance, we have drawn a parallel between squash (Marino et al., 2010) and pumpkin used in this study due to their similarity in chemical composition (Paris and Brown, 2005). Therefore, it can be inferred that an increase in% CH4 in the gas sample led to an increase in CH4 production (mg/g DM) because CH_4_ production = CH_4_ (%) × gas production (mL/g DM).

It is important to note that an increase in the level of vegetable on a DM basis led to an increase in sorghum silage IVDMD (Table 3). There is considerable evidence in the literature demonstrating a negative relationship of NDF content and IVDMD. Increased levels of vegetables in the silage decreased the aNDFom concentration thereby increasing IVDMD. Consequently, this effect was directly associated with a greater concentration of total VFA produced during rumen fermentation.

## Conclusion

Overall, incorporation of vegetables in a sorghum crop silage at either 20 or 40% on a DM basis positively influenced the crude fat content and VFA concentrations, despite a minor decrease in fiber content. Further, the use of carrots at 40% DM in the silage enhanced bacterial and richness and diversity. Upon aerobic exposure, vegetable inclusion also at 40% DM increased fungal richness and diversity. Moreover, incorporation of 20% carrot or pumpkin in the silage DM was an optimal proportion to minimize CH_4_ production parameters, increase digestibility and increase total concentration of VFA. Consequently, this study has identified the potential for unsalable vegetables to be ensiled with crop sorghum and is therefore conducive to the production of a sustainable, high-quality alternative ruminant feed.

## Data Availability Statement

The datasets generated for this study can be found in Sequence Read Archive under BioProject, PRJNA525436.

## Ethics Statement

Steers used in this study were cared for under the approval and guidance of The University of Queensland Animal Ethics Committee (Approved Protocol Number AE35581).

## Author Contributions

AC and SM designed the study. AC, SM, DF, KH, EC, YH, and DH acquired data, read and critically revised drafts for intellectual contents, and approved the final manuscript. EC, DF, KH, YH, and AC conducted laboratory analysis. DH conducted bioinformatics. AC and DH ran statistical analysis. DF, KH, DH, SM, and AC wrote the manuscript.

## Conflict of Interest

The authors declare that the research was conducted in the absence of any commercial or financial relationships that could be construed as a potential conflict of interest.
