# Supplementary material for: Crop Sorghum Ensiled With Unsalable Vegetables Increases Silage Microbial Diversity
Source: Front Microbiol. 2019 Nov 15;10:2599. doi: 10.3389/fmicb.2019.02599 (PMC6872954; doi:10.3389/fmicb.2019.02599)
Supplement: Supplementary file 1 [file Table_1.DOCX]

| **Supplementary Table 1.** Differentially abundant OTUs in the bacterial microbiota of either the 100% sorghum silage and 40% carrot or 100% sorghum and 40% pumpkin silage at sampling day 1 and 2. BaseMean represents the normalized OTU abundance among all samples compared. Negative log2 fold change values indicate lower abundance in the 100% sorghum silage. Only differentially abundant OTUs with an adjusted p-value of less than 0.01 are included. | | | | |
| --- | --- | --- | --- | --- |
|  |  |  |  |  |
| **OTU ID** | **BaseMean** | **log2 fold change** | **Adjusted p-value** | **Genus** |
| **100% Sorghum vs. 40% Carrot - Sampling day 1** |  |  |  |  |
| OTU34 | 184.25 | -24.84 | 1.71E-11 | *Pseudomonas* |
| OTU14 | 80.13 | -23.71 | 2.47E-11 | *Lactobacillus* |
| OTU74 | 99.24 | -22.27 | 4.66E-10 | *Lactobacillus* |
| OTU45 | 343.40 | -11.36 | 1.82E-03 | *Lactobacillus* |
| OTU26 | 214.79 | -10.68 | 3.64E-03 | *Lactobacillus* |
| OTU62 | 122.01 | -9.86 | 1.82E-03 | *Brucella* |
| OTU44 | 52.66 | -8.65 | 5.51E-03 | *Lactobacillus* |
| **100% Sorghum vs. 40% Carrot - Sampling day 2** |  |  |  |  |
| OTU34 | 451.05 | -25.49 | 1.29E-12 | *Pseudomonas* |
| OTU52 | 318.90 | -24.92 | 2.23E-12 | *Achromobacter* |
| OTU32 | 169.37 | -24.23 | 4.94E-12 | *Lactobacillus* |
| OTU55 | 226.57 | -24.17 | 1.78E-11 | *Rummeliibacillus* |
| OTU31 | 70.50 | -23.01 | 3.85E-11 | *Lactobacillus* |
| OTU69 | 129.32 | -22.49 | 1.09E-10 | *Olivibacter* |
| OTU86 | 33.79 | -21.98 | 3.89E-10 | *Gluconobacter* |
| OTU56 | 50.19 | -20.92 | 3.03E-09 | *Stenotrophomonas* |
| OTU7 | 101.68 | -9.85 | 5.80E-03 | *Lactobacillus* |
| OTU35 | 76.35 | -9.43 | 1.25E-03 | *Lactobacillus* |
| OTU44 | 48.68 | -8.78 | 3.80E-03 | *Lactobacillus* |
| OTU78 | 117.61 | 24.81 | 1.29E-12 | *Lactobacillus* |
| **100% Sorghum vs. 40% Pumpkin - Sampling day 1** |  |  |  |  |
| OTU35 | 131.09 | -10.34 | 1.31E-04 | *Lactobacillus* |
| OTU10 | 71.99 | 24.28 | 5.10E-14 | *Lactobacillus* |
| OTU41 | 219.36 | 25.80 | 1.33E-14 | *Lactobacillus* |
| OTU59 | 317.81 | 26.30 | 9.14E-15 | *Lachnoclostridium* |
| **100% Sorghum vs. 40% Pumpkin - Sampling day 2** |  |  |  |  |
| OTU122 | 145.77 | -25.10 | 1.00E-16 | *Lactobacillus* |
| OTU31 | 81.95 | -24.29 | 1.38E-16 | *Lactobacillus* |
| OTU134 | 38.84 | -9.57 | 4.34E-03 | *Pseudomonas* |
| OTU60 | 31.90 | -9.29 | 4.34E-03 | *Megasphaera* |

**Supplementary Figure S1.** Temperature difference between the environment and on the sorghum silage surface upon aerobic exposure from day 1 to day 14.

^1^ P-values for the treatments “Vegetable” (i.e. carrot or pumpkin) and “Vegetable × Level” interaction P>0.10. “Level” (i.e. proportion of vegetable in silage on DM basis) P≤0.05. ^a-c^Bars with different superscripts within each day differ at P≤0.05.

**Supplementary Figure S2.** Non-metric dimensional scaling (NMDS) plot of the Bray-Curtis dissimilarities for the initial bacterial microbiota by vegetable mixture, sampling day, and use of an inoculant (stress = 0.06).

**Supplementary Figure S3.** The 10 most relatively abundant bacterial genera in the initial silage microbiota by ensiled vegetable mixture of carrot or pumpkin at 0%, 20% or 40% DM. Different lowercase letters indicate significantly different means (P≤0.05).
