# Supplementary figures and images for: Crop Sorghum Ensiled With Unsalable Vegetables Increases Silage Microbial Diversity
Source: Front Microbiol. 2019 Nov 15;10:2599. doi: 10.3389/fmicb.2019.02599 (PMC6872954; doi:10.3389/fmicb.2019.02599)

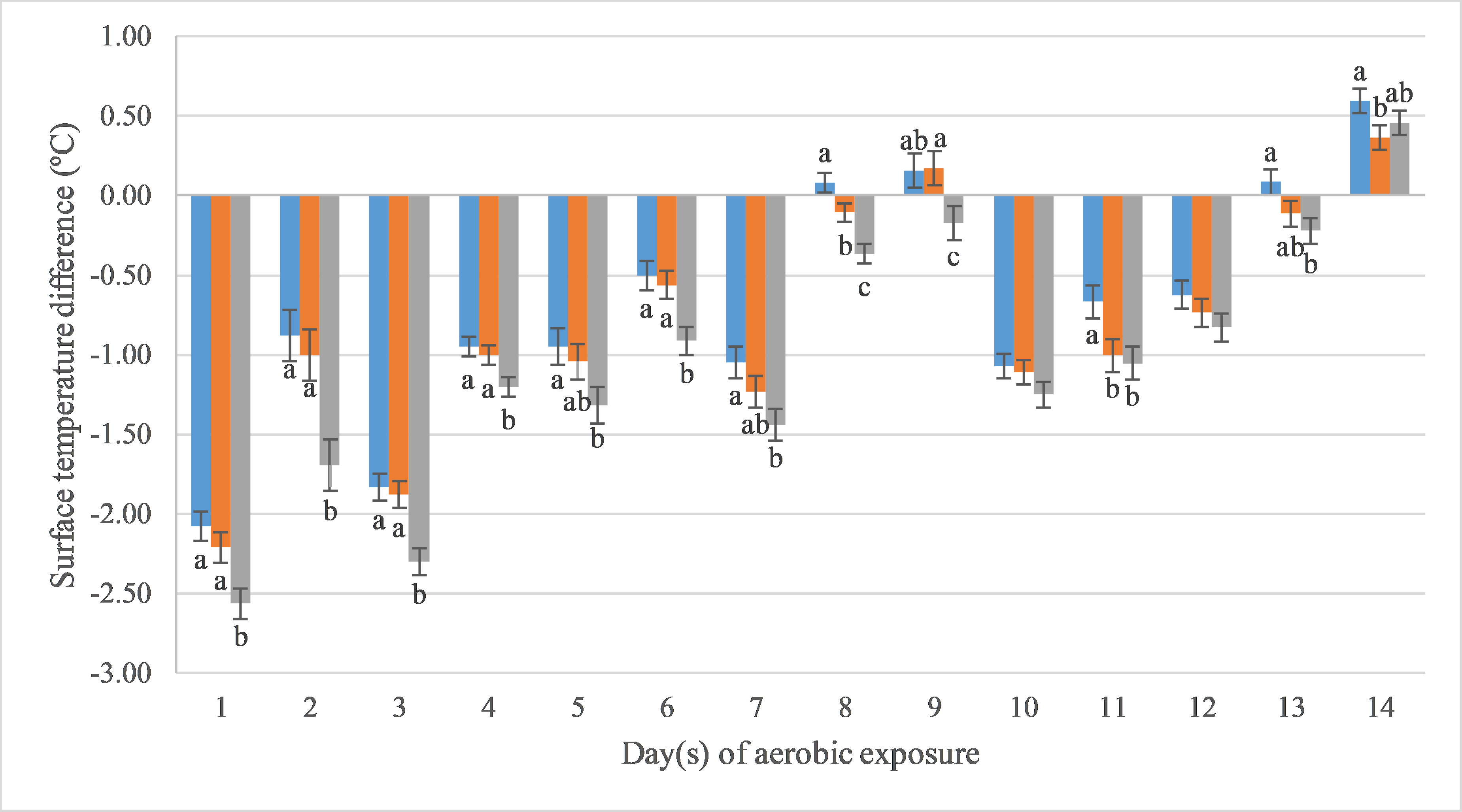

Supplement: Supplementary file 2 [file Image_1.TIF]

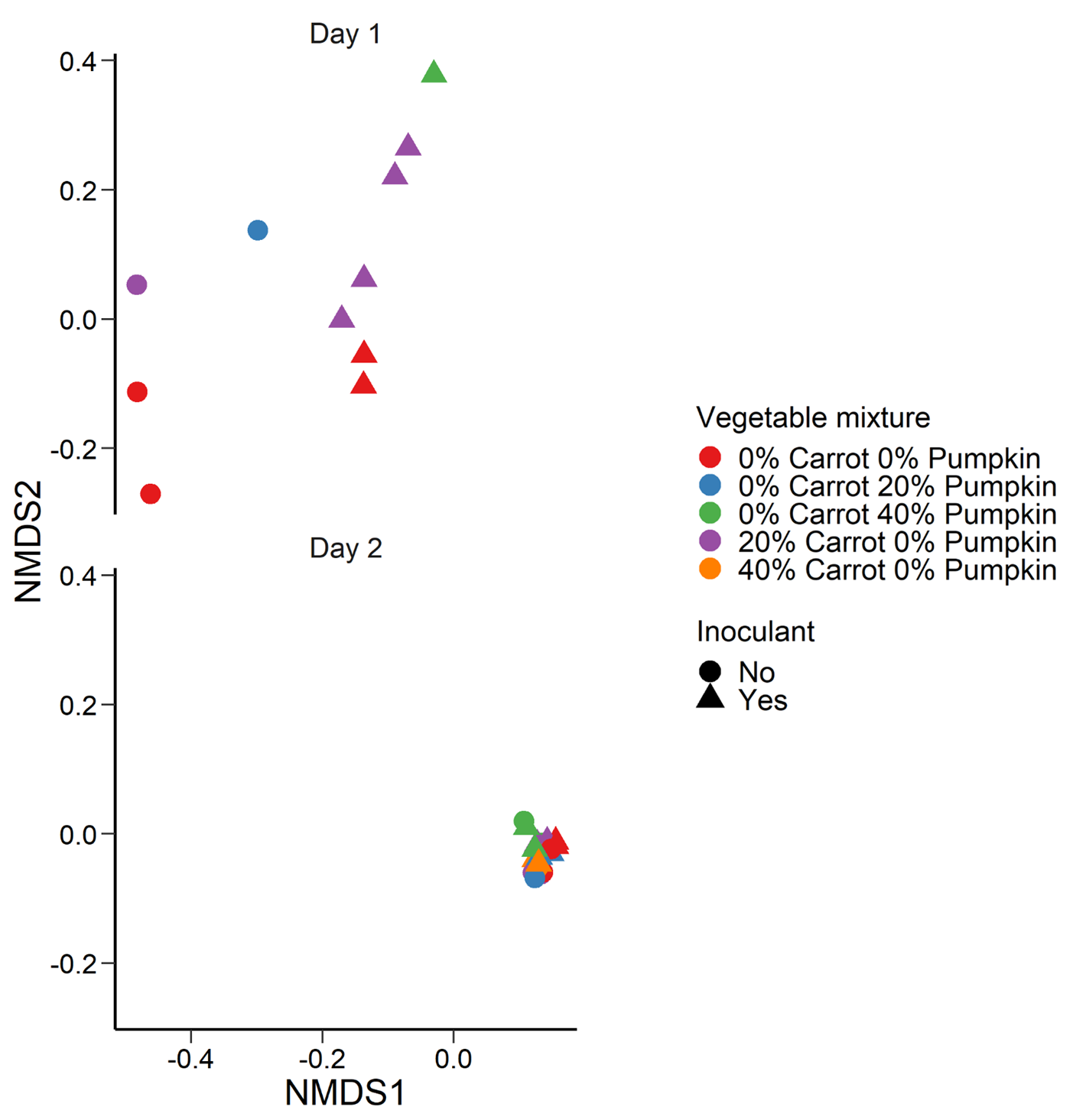

Supplement: Supplementary file 3 [file Image_2.TIFF]

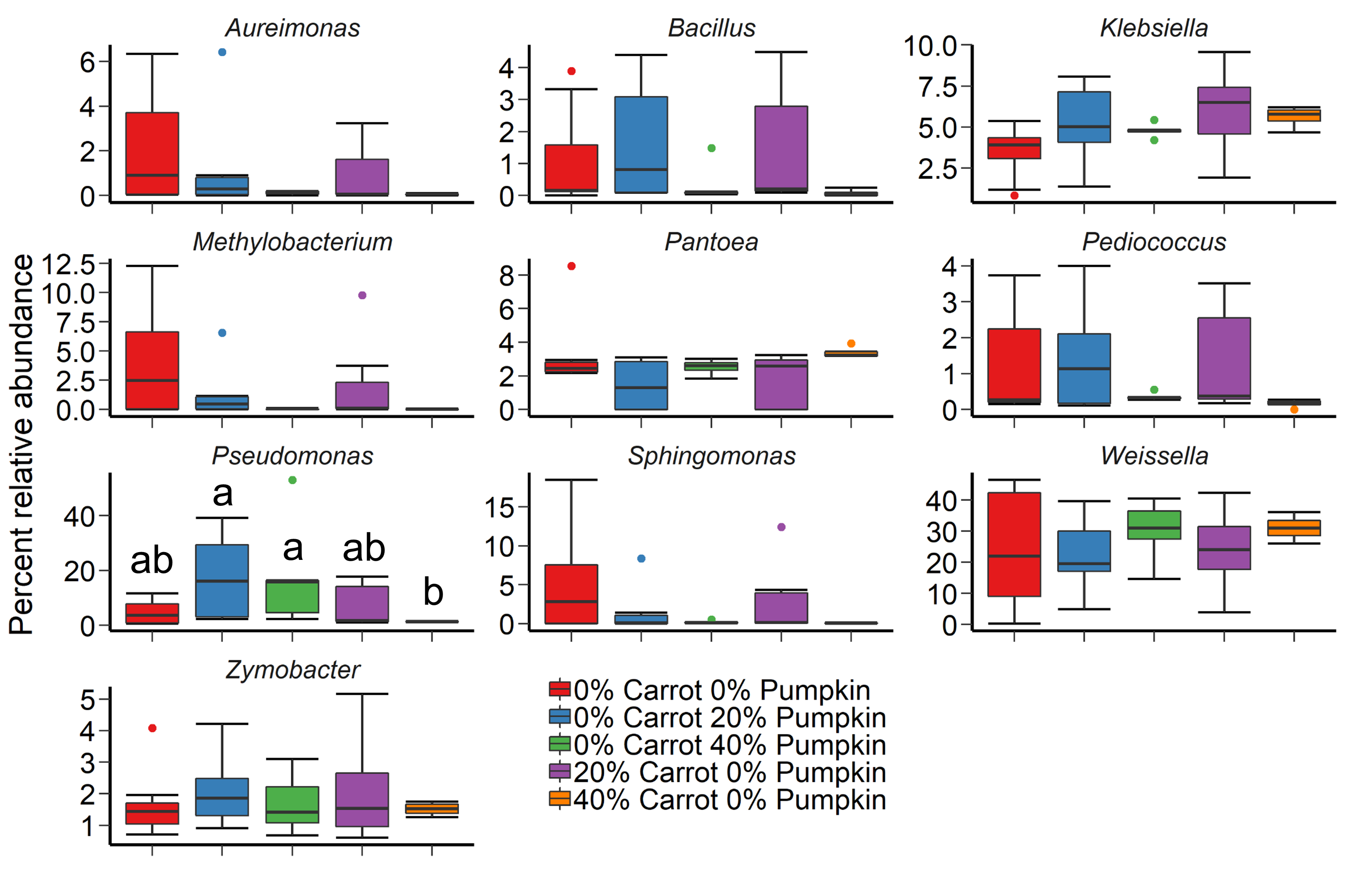

Supplement: Supplementary file 4 [file Image_3.TIFF]
